# Thrombotic complications in critically ill patients with coronavirus infections: A comparative study of MERS and COVID-19

**DOI:** 10.1097/MD.0000000000046210

**Published:** 2025-11-21

**Authors:** Mosaad Almegren, Turki Alshuaibi, Amal M. Alhusayni, Abdulrahman Alraizah, Rayan Qutob, Bader Al Rawahi, Eysa Alsolamy, Fatima Sharif

**Affiliations:** a Imam Mohammad ibn Saud Islamic University, Riyadh, Saudi Arabia; b King Fahad Hospital, Jeddah, Saudi Arabia; c King Abdulaziz Medical City, Riyadh, Saudi Arabia; d Sultan Qaboos University Hospital, Muscat, Oman; e Medi Test Lab and Diagnostic Center, Islamabad, Pakistan.

**Keywords:** COVID-19, critical care, MERS-CoV, thrombosis

## Abstract

Coronaviruses include coronavirus disease 2019 (COVID-19) and Middle East respiratory syndrome (MERS). These viruses cause respiratory infections in humans and share several overlapping structural, pathological, and genetic features. This study compared arterial and venous thrombotic events in critically ill patients with COVID-19 and MERS. This study was conducted in the intensive care units of 2 hospitals in Saudi Arabia. Polymerase chain reaction-positive patients who were diagnosed with COVID-19 between February and July 2020 and polymerase chain reaction-positive patients with MERS from March to May 2014 were included. The data were analyzed via SAS version 9.4 (SAS Institute, Cary, NC). This study included 294 patients, 234 (79.6%) of whom had COVID-19, and 60 (20.4%) had MERS. Overall, the rate of composite thrombotic events was 44 (14.97%) in both groups, comprising 25 patients with venous thromboembolism (23 patients with COVID-19 and 2 in the MERS group) and 19 with arterial thrombotic episodes (14 patients with COVID-19 and 5 in the MERS group). Bronchial asthma was significantly associated with thrombosis (*P* = .02). Enoxaparin was the most frequently prescribed thromboprophylaxis 189 (64.5%). There was no significant difference in venous thromboembolism (*P* = .10), bleeding complications (*P* = .53), or mortality (*P* = .08) between the COVID-19 and MERS patients. We found a similar rate of composite thrombotic events in critically ill patients with COVID-19 and MERS. While the thrombotic tendency of patients with COVID-19 was previously known, we uniquely identified the role of MERS in both arterial and venous thrombosis.

## 1. Introduction

Coronaviruses are single-stranded RNA viruses that infect animals and humans, causing respiratory infections.^[1]^ Some well-known pathogenic coronaviruses implicated in human infections include severe acute respiratory syndrome (SARS) and SARS-coronavirus 2 (CoV-2), also known as coronavirus disease 2019 (COVID-19) and Middle East respiratory syndrome (MERS).^[2]^

COVID-19 emerged in China in December 2019 and spread worldwide, with 776 million cases documented as of November 2024.^[3]^ While it is predominantly a respiratory infection, COVID-19 is associated with a variety of signs and symptoms ranging from asymptomatic mild infection to severe pneumonia, as well as extrapulmonary manifestations involving the gastrointestinal and neurological systems.^[4,5]^ COVID-19 has also had a profound impact on the blood coagulation system.^[6]^ Klok et al reported the occurrence of venous thromboembolism (VTE) in 27% of intensive care unit (ICU) patients with COVID-19.^[7]^ A multicenter study from Saudi Arabia also reported a high rate of arterial thrombotic events, reaching 2.2% in critically ill COVID-19 patients.^[8]^

MERS-CoV was initially reported in Saudi Arabia in 2012, with 2613 confirmed cases reported globally to the World Health Organization from 2012 to May 2024.^[9]^ MERS is a severe respiratory infection with a high risk of mortality (55%).^[10]^ However, the risk of thrombotic complications in MERS patients has not been established in the literature to date.

Several common features of COVID-19 and MERS, including transmissibility and clinical presentation, have been identified.^[11]^ Molecular analysis has shown that there is approximately 50% similarity between the genomic structures of COVID-19 and MERS.^[12]^ Clinically, COVID-19 and MERS show similar involvement of the pulmonary and cardiac systems and nonspecific gastrointestinal complications; however, hematological complications, especially hypercoagulability, are notoriously associated with COVID-19.^[13]^

While the association between COVID-19 and thrombosis is well established, the association between MERS and thrombosis is not well-known. As both viruses share many overlapping features, MERS may be a causative agent of thrombosis. Therefore, this bicenter study aimed to compare the rate of thrombotic events in critically ill patients hospitalized with COVID-19 or MERS.

## 2. Materials and methods

This retrospective study was conducted in 2 major hospitals in the central and western regions of Saudi Arabia. We included all confirmed COVID-19 patients admitted to the ICU between February and July 2020. A standardized electronic form comprising demographics, comorbidities, type and dose of thromboprophylaxis, thrombotic events, bleeding events, and laboratory results was designed for data collection. Data were collected retrospectively for adult patients ≥18 years from the first day of ICU admission to discharge, end of study period or death. Patients were excluded if they were transferred to another hospital, admitted for less than 24 hours, or if the required data were incomplete.

A confirmed positive COVID-19 case was defined as a positive reverse-transcriptase polymerase chain reaction test via an oral or nasopharyngeal swab. We also retrospectively included all confirmed cases of MERS-CoV diagnosed via polymerase chain reaction in patients admitted to the ICU between March and May 2014.

The Padua prediction score is a validated instrument used to evaluate the risk of VTE.^[14]^ This score incorporates factors such as age, active malignancy, previous thrombosis, immobility, and recent trauma/surgery and assesses the impact of these risk factors on the occurrence of thrombosis.^[14]^

VTE and arterial thrombosis were radiologically confirmed. Investigations for VTE, including Doppler ultrasound of the extremities or computed tomography pulmonary angiography, were performed if there was clinical suspicion of the disease. No screening for asymptomatic VTE was performed. Cerebrovascular accident was confirmed by brain magnetic resonance imaging or computed tomography of the brain, whereas myocardial infarction (MI) was confirmed by elevated cardiac enzymes and electrocardiogram.

The primary outcome of this study was the frequency of VTE, including PE, DVT, and unusual vein thrombosis. Other outcomes included the frequency of arterial thrombotic events, composite outcomes (venous and arterial thrombosis), and additional complications, including bleeding and mortality. Hemorrhagic complications were defined as major or nonmajor complications based on the standardized criteria recommended by the International Society of Thrombosis and Hemostasis.^[15]^ According to the guidelines, major bleeding events are those episodes that result in mortality, cause a drop of 2 g/dL hemoglobin, and/or require at least 2 units of whole blood or red cells, or bleeding that occurs in critical organs, such as the intracranial and intrapericardial areas. All other hemorrhagic episodes were categorized as nonmajor.^[15]^ Patient outcomes were assessed until discharge from the hospital or death.

Data were entered and analyzed using SAS version 9.4 (SAS Institute, Cary, NC). Means and standard deviations were calculated for quantitative variables such as age and laboratory parameters, whereas frequencies and percentages were reported for qualitative variables such as comorbidities, occurrence of VTE, and mortality. Odds ratios with 95% confidence intervals were determined for the risk factors associated with the development of VTE in patients with MERS and COVID-19. Kaplan–Meier curves were plotted for survival analysis. Statistical significance was set at *P* < .05.

The study protocol was approved by the institutional review board at King Fahad Hospital (A01685), and informed consent was waived due to the nature of the study. The confidentiality of all study participants was maintained. All methods were performed in accordance with relevant guidelines and regulations.

## 3. Results

This study included 294 patients, 234 (79.6%) diagnosed with COVID-19 and 60 (20.4%) with MERS infection. The majority (n = 225, 76.5%) of the patients were male. The mean age of our patients was 58.9 ± 17.1 years.

Hypertension was the most common underlying comorbidity in 154 patients (52.4%), followed by diabetes mellitus in 144 patients (49%). The mean baseline Padua score was 2.6 ± 1.78, with 75 (25.6%) patients having a Padua score ≥ 4, indicating a high risk of VTE. Among the laboratory parameters, the baseline activated partial thromboplastin time was significantly higher in patients with MERS than in those with COVID-19 (*P* < .0001) (Table 1).

**Table 1:** Summarizes the patients’ baseline demographic characteristics.

Characteristic	All patients (N = 294)	ICU COVID (N = 234)	ICU MERS (N = 60)	*P* value
Age (yr), mean ± SD	58.9 ± 17.1	62.2 ± 15.02	45.8 ± 18.68	**<.001**
Males N (%)	225 (76.5)	181 (80.4)	44 (73.3)	.512
Weight (kg), mean ± SD	81.5 ± 18.9	82.5 ± 20.23	77.5 ± 12.18	.265
Comorbidities				
Diabetes, n (%)	144 (49)	134 (57.3)	10 (16.7)	**<.001**
Hypertension, n (%)	154 (52.4)	134 (57.3)	20 (33.3)	**<.001**
Coronary artery disease, n (%)	30 (10.2)	26 (11.1)	4 (6.7)	.310
Stroke, n (%)	15 (5.1)	15 (6.4)	0	.047
COPD, n (%)	8 (2.7)	7 (3.0)	1 (1.67)	1.000
Bronchial asthma, n (%)	18 (6.1)	17 (7.3)	1 (1.67)	.136
Heart failure, n (%)	20 (6.8)	18 (7.7)	2 (3.33)	.387
Previous DVT/PE, n (%)	9 (3.1)	9 (3.8)	0	.212
Active cancer, n (%)	9 (3.1)	9 (3.8)	0	.212
ESRD, n (%)	15 (5.1)	9 (3.8)	6 (10)	.091
Padua score, mean ± SD	2.6 ± 1.78	2.9 ± 1.87	1.5 ± 0.57	**<.001**
Padua categorized				**<.001**
<4, n (%)	218 (74.4)	158 (67.5)	60 (100)	
≥4, n (%)	75 (25.6)	75 (32.1)	0	
Platelet count ×10^9^/L, mean ± SD	266.6 ± 129.54	265.1 ± 109.34	272 ± 189.72	.789
Prothrombin time, mean ± SD	13.3 ± 7.78	13.2 ± 8.38	13.8 ± 5.03	.377
Activated partial thromboplastin time, mean ± SD	36.7 ± 26.03	32.2 ± 9.9	53 ± 49.93	**<.0001**
d dimer (mg/L), mean ± SD	5 ± 8.5	5.10 ± 8.62	3.8 ± 2.75	.276

The bold values indicate *P* < .05.

**Figure 1.**
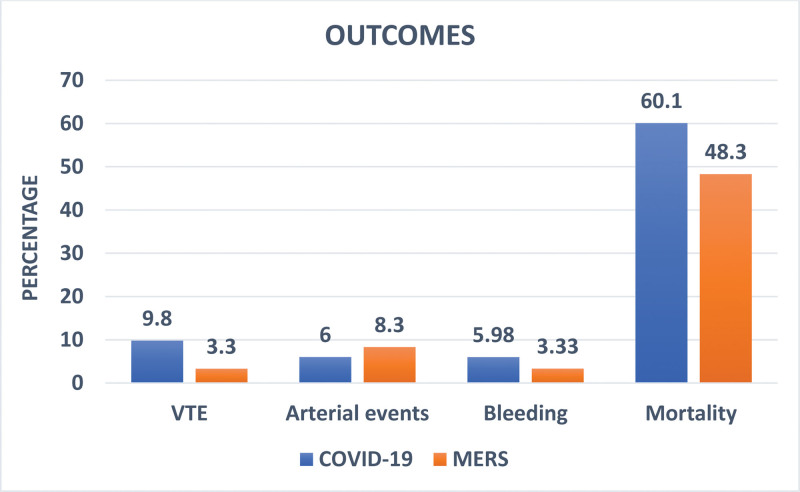
Comparison of the frequency of thrombosis, bleeding and mortality among patients with COVID-19 and MERS.

Overall, the incidence of thrombotic events, including both arterial and venous thromboses, was 44 (14.9%) in our study population. Twenty-five (8.6%) of the study population developed VTE, which comprised 23 of 234 COVID-19 patients (9.8%) and 2 of 60 MERS patients (3.3%). There was no statistically significant difference in the incidence of VTE between COVID-19 and MERS patients (*P* = .10). Furthermore, 19 patients (6.4%) experienced arterial episodes, including MI, Cerebrovascular accident and limb ischemia. These patients comprised 14 of 234 COVID-19 patients (6.0%) and 5 of 60 MERS patients (8.3%) (*P* = .55). All thromboses (venous and arterial) were similar between groups (*P* = .62). See Figure 1.

The median follow-up period was 21 days (range: 1–226 days). The median duration from ICU admission to the development of VTE was 17 days (range: 0–159 days) for COVID-19 patients and 11.5 days (range: 0–23 days) for those with MERS (*P* = .46). The median time from ICU admission to the occurrence of arterial events was 6 days (range: 0–27 days) for COVID-19 patients and 0 days (range: 0–3 days) for MERS patients (*P* = .046).

Most COVID-19 patients 98% (n = 229) and 67% (n = 40) of MERS patients were on thromboprophylaxis (*P* < 001). Unfractionated heparin was administered to 80 (27.2%) patients, whereas 189 (64.5%) patients received enoxaparin.

A total of 16 patients (5.4%) experienced bleeding episodes as a complication of anticoagulation, including 7 patients with major bleeding, as defined by the International Society of Thrombosis and Hemostasis criteria, and 9 patients with nonmajor bleeding events. There was no statistically significant difference in the incidence of any bleeding events among patients with COVID-19 (14 of 234 patients, 5.98%) or MERS (2 of 60 patients, 3.33%) (*P* = .53). Hundred seventy-one out of the total 294 patients (58.2%) died during the study period. These included 142 out of 234 (60.1%) patients with COVID-19 and 29 out of 60 patients (48.3%) with MERS (*P* = .08). There was no difference in the mortality rate between patients with and without thrombosis (*P* = .65). See Figure 1.

On multivariate logistic regression analysis, bronchial asthma was identified as the only underlying condition significantly associated with the development of thrombosis, including both VTE and arterial events (odds ratios, 3.9; 95% CI: 1.17–12.98; *P* = .02).

**Figure 2.**
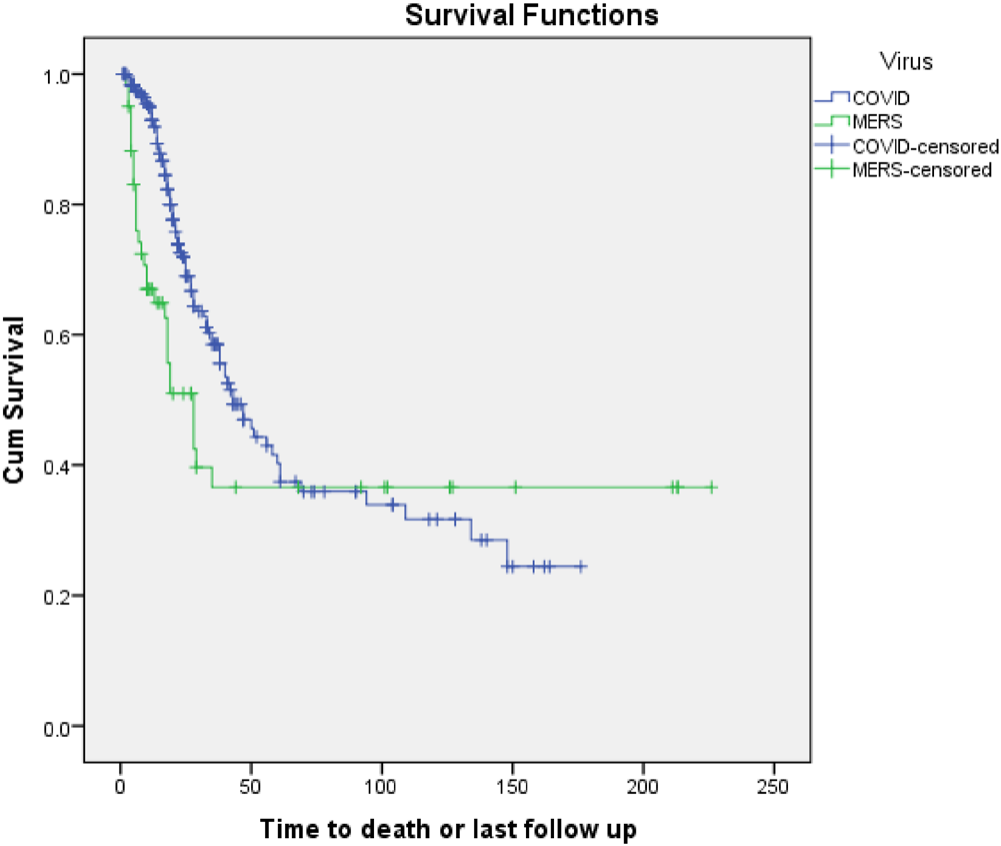
Comparison of median survival for COVID and MERS in all patients with thrombosis.

According to the Kaplan–Meier survival analysis, the median overall survival of all 234 patients with COVID-19 was 34 days, whereas it was 68 days for all 60 patients with MERS (*P* = .07). Among patients with thrombosis (arterial or venous), the median survival of COVID-19 patients was 42 days, whereas that of MERS patients with thrombosis was 68 days (*P* = .47). Figure 2 shows the Kaplan–Meier curves with survival analysis of patients who experienced thrombotic events, stratified according to the underlying viral infection status.

## 4. Discussion

This study of 294 patients assessed the incidence of venous or arterial thrombosis in critically ill patients with COVID-19 and MERS along with their outcomes and impact on survival. The overall composite thrombotic event rate was 14.9%. We found a similar incidence of composite thrombotic episodes (venous or arterial) between critically ill patients with COVID-19 and MERS.

Almost 10% of COVID-19 patients develop VTE. The frequency of VTE in patients with COVID-19 has been highly variable in the literature, ranging from 3.1% to 85.4% in different patient populations.^[16,17]^ Approximately 6% of COVID-19 patients experience arterial thrombotic episodes including stroke and MI. This percentage is higher than that reported in previous studies that reported a relatively low frequency of arterial thrombotic episodes in patients with COVID-19, ranging from 0.8% to 4.4%.^[18,19]^

Among the 60 patients infected with MERS, 3.3% experienced VTE, and 8.3% experienced arterial thrombotic episodes. While the clinical association between MERS and thrombosis has not been previously reported, its pathogenesis was studied in mouse models by Li et al in 2016.^[20]^ Histological examination revealed microthrombi in the pulmonary vasculature of transgenic mice expressing human dipeptidyl peptidase 4 (hDPP4), the receptor for MERS entry into human cells.^[20]^ Among our patients, MERS patients suffered from episodes of arterial ischemia more frequently than COVID-19 patients (8.3% vs 6%); however, this difference was not statistically significant.

COVID-19 and MERS have many comparable and contrasting features, in terms of their biological structures and clinical implications. The M protein (membrane protein), which is considered a crucial building block of the virus structure, is similar in both viruses and comprises polylactosamine chains.^[21]^ However, the spike proteins of both viruses are significantly different, which influences their transmissibility.^[22]^ In terms of coagulation abnormalities, both COVID-19 and MERS are associated with thrombocytopenia and a propensity for disseminated intravascular coagulation.^[23]^ In our study, we demonstrated a similar tendency for arterial and venous thromboses.

In multivariate logistic regression analysis, bronchial asthma was the only statistically significant factor associated with the occurrence of thrombosis in our patients. A nationwide cohort study from Taiwan also revealed that asthma is significantly associated with pulmonary embolism, with a hazard ratio of 3.24 compared with non-asthmatic patients.^[24]^

Overall mortality was significantly greater in COVID-19 patients than in those with MERS. In contrast, a meta-analysis of 114 articles that included MERS and COVID‐19 patients revealed that the overall case fatality rate was significantly higher in MERS patients than in COVID-19 patients (35% vs 5.6%, *P* < .001).^[25]^ However, patients with COVID-19 and MERS, who experienced thrombotic events, had similar survival rates.

To the best of our knowledge, this is among the first comparisons of thrombotic episodes between patients with COVID-19 and MERS. The association between VTE and COVID-19 has already been well established, but the role of MERS in thrombosis was uniquely identified in this study. However, the number of MERS patients included in this study was limited. Our findings need to be confirmed on a larger scale, with standardization of thromboprophylactic regimens used so that the direct impact of COVID-19 and MERS on thrombosis may be assessed with minimum possible confounding factors.

## 5. Conclusion

We found a similar incidence of composite thrombotic episodes among critically ill patients with COVID-19 and MERS, with VTE being more common in COVID-19 patients and arterial thrombosis being more common in MERS patients; however, the difference was not statistically significant. Although the impact of COVID-19 on thrombosis is well-known, our findings highlight that MERS may also be an important cause of thrombosis, similar to COVID-19.

## Acknowledgments

We used Paperpal AI for English language editing services.

## Author contributions

**Conceptualization:** Mosaad Almegren.

**Data curation:** Mosaad Almegren, Turki Alshuaibi, Amal M. Alhusayni, Abdulrahman Alraizah, Rayan Qutob, Eysa Alsolamy, Fatima Sharif.

**Formal analysis:** Bader Al Rawahi, Fatima Sharif.

**Project administration:** Mosaad Almegren.

**Supervision:** Mosaad Almegren.

**Writing – original draft:** Mosaad Almegren, Fatima Sharif.

**Writing – review & editing:** Mosaad Almegren, Turki Alshuaibi, Amal M. Alhusayni, Abdulrahman Alraizah, Rayan Qutob, Bader Al Rawahi, Eysa Alsolamy.

## References

[1] Weiss SR Leibowitz JL. Coronavirus pathogenesis. Adv Virus Res. 2011;81:85–164. 22094080 10.1016/B978-0-12-385885-6.00009-2 PMC7149603

[2] Kesheh MM Hosseini P Soltani S Zandi M. An overview on the seven pathogenic human coronaviruses. Rev Med Virol. 2022;32:e2282. 34339073 10.1002/rmv.2282

[3] https://data.who.int/dashboards/covid19/cases. Accessed October 15, 2024.

[4] Xu E Xie Y Al-Aly Z. Long-term gastrointestinal outcomes of COVID-19. Nat Commun. 2023;14:983. 36882400 10.1038/s41467-023-36223-7 PMC9992516

[5] Ermis U Rust MI Bungenberg J. Neurological symptoms in COVID-19: a cross-sectional monocentric study of hospitalized patients. Neurol Res Pract. 2021;3:1–2. 33712089 10.1186/s42466-021-00116-1 PMC7953515

[6] Luo HC You CY Lu SW Fu YQ. Characteristics of coagulation alteration in patients with COVID-19. Ann Hematol. 2021;100:45–52. 33079220 10.1007/s00277-020-04305-x PMC7572245

[7] Klok FA Kruip MJ Van der Meer NJ. Incidence of thrombotic complications in critically ill ICU patients with COVID-19. Thromb Res. 2020;191:145–7. 32291094 10.1016/j.thromres.2020.04.013 PMC7146714

[8] Al Raizah A Al Askar A Shaheen N. High rate of bleeding and arterial thrombosis in COVID-19: Saudi multicenter study. Thromb J. 2021;19:13. 33658062 10.1186/s12959-021-00265-y PMC7928187

[9] https://www.emro.who.int/health-topics/mers-cov/mers-outbreaks.html. Accessed October 15, 2024.

[10] Assiri A Al-Tawfiq JA Al-Rabeeah AA. Epidemiological, demographic, and clinical characteristics of 47 cases of Middle East respiratory syndrome coronavirus disease from Saudi Arabia: a descriptive study. Lancet Infect Dis. 2013;13:752–61. 23891402 10.1016/S1473-3099(13)70204-4 PMC7185445

[11] Petrosillo N Viceconte G Ergonul O Ippolito G Petersen E. COVID-19, SARS and MERS: are they closely related? Clin Microbiol Infect. 2020;26:729–34. 32234451 10.1016/j.cmi.2020.03.026 PMC7176926

[12] Lu R Zhao X Li J. Genomic characterization and epidemiology of 2019 novel coronavirus: implications for virus origins and receptor binding. Lancet. 2020;395:565–74. 32007145 10.1016/S0140-6736(20)30251-8 PMC7159086

[13] Gerges Harb J Noureldine HA Chedid G. SARS, MERS and COVID-19: clinical manifestations and organ-system complications: a mini review. Pathog Dis. 2020;78:ftaa033. 32633327 10.1093/femspd/ftaa033 PMC7454523

[14] Zeng DX Xu JL Mao QX. Association of Padua prediction score with in-hospital prognosis in COVID-19 patients. QJM. 2020;113:789–93. 32652021 10.1093/qjmed/hcaa224 PMC7454846

[15] Schulman S Kearon C. Definition of major bleeding in clinical investigations of antihemostatic medicinal products in non‐surgical patients. J Thromb Haemost. 2005;3:692–4. 15842354 10.1111/j.1538-7836.2005.01204.x

[16] Hill JB Garcia D Crowther M. Frequency of venous thromboembolism in 6513 patients with COVID-19: a retrospective study. Blood Adv. 2020;4:5373–7. 33137202 10.1182/bloodadvances.2020003083 PMC7656921

[17] Ren B Yan F Deng Z. Extremely high incidence of lower extremity deep venous thrombosis in 48 patients with severe COVID-19 in Wuhan. Circulation. 2020;142:181–3. 32412320 10.1161/CIRCULATIONAHA.120.047407

[18] Burn E Duarte-Salles T Fernandez-Bertolin S. Venous or arterial thrombosis and deaths among COVID-19 cases: a European network cohort study. Lancet Infect Dis. 2022;22:1142–52. 35576963 10.1016/S1473-3099(22)00223-7 PMC9106320

[19] Cheruiyot I Kipkorir V Ngure B Misiani M Munguti J Ogeng'o J. Arterial thrombosis in coronavirus disease 2019 patients: a rapid systematic review. Ann Vasc Surg. 2021;70:273–81. 32866574 10.1016/j.avsg.2020.08.087 PMC7453204

[20] Li K Wohlford-Lenane C Perlman S. Middle East respiratory syndrome coronavirus causes multiple organ damage and lethal disease in mice transgenic for human dipeptidyl peptidase 4. J Infect Dis. 2016;213:712–22. 26486634 10.1093/infdis/jiv499 PMC4747621

[21] Juckel D Desmarets L Danneels A Rouillé Y Dubuisson J Belouzard S. MERS-CoV and SARS-CoV-2 membrane proteins are modified with polylactosamine chains. J Gen Virol. 2023;104:001900. 10.1099/jgv.0.001900 37800895

[22] D’Arco A Di Fabrizio M Mancini T. Secondary structures of MERS-CoV, SARS-CoV, and SARS-CoV-2 spike proteins revealed by infrared vibrational spectroscopy. Int J Mol Sci. 2023;24:9550. 37298500 10.3390/ijms24119550 PMC10253540

[23] Giannis D Ziogas IA Gianni P. Coagulation disorders in coronavirus infected patients: COVID-19, SARS-CoV-1, MERS-CoV and lessons from the past. J Clin Virol. 2020;127:104362. 32305883 10.1016/j.jcv.2020.104362 PMC7195278

[24] Chung WS Lin CL Ho FM. Asthma increases pulmonary thromboembolism risk: a nationwide population cohort study. Eur Respir J. 2014;43:801–7. 23988762 10.1183/09031936.00043313

[25] Pormohammad A Ghorbani S Khatami A. Comparison of confirmed COVID‐19 with SARS and MERS cases – clinical characteristics, laboratory findings, radiographic signs and outcomes: a systematic review and meta‐analysis. Rev Med Virol. 2020;30:e2112. 32502331 10.1002/rmv.2112 PMC7300470

